# The Role of Antibody Testing for SARS-CoV-2: Is There One?

**DOI:** 10.1128/JCM.00797-20

**Published:** 2020-07-23

**Authors:** Elitza S. Theel, Patricia Slev, Sarah Wheeler, Marc Roger Couturier, Susan J. Wong, Kamran Kadkhoda

**Affiliations:** a Division of Clinical Microbiology, Department of Laboratory Medicine and Pathology, Mayo Clinic, Rochester, Minnesota, USA; b ARUP Laboratories, Salt Lake City, Utah, USA; c Department of Pathology, University of Utah School of Medicine, Salt Lake City, Utah, USA; d Division of Clinical Immunopathology, Department of Pathology, University of Pittsburgh, Pittsburgh, Pennsylvania, USA; e Wadsworth Center, New York State Department of Health, Albany, New York, USA; f Immunopathology Laboratory, Robert J. Tomsich Pathology and Laboratory Medicine Institute, Cleveland Clinic, Cleveland, Ohio, USA; Boston Children's Hospital

**Keywords:** antibody, COVID-19, SARS-CoV-2, serology

## Abstract

The emergence of severe acute respiratory syndrome coronavirus 2 (SARS-CoV-2) brought with it rapid development of both molecular and serologic assays for identification of COVID-19 infections. While Food and Drug Administration (FDA) emergency use authorization (EUA) is required for clinical application of SARS-CoV-2 molecular tests, submission for EUA is currently a voluntary process for manufacturers of serologic assays. The absence of FDA oversight of serologic tests is concerning given that the commercially available serologic assays are highly variable, differing in their format, the antibody class detected, the targeted antigen, and the acceptable specimen types. An added complication is the lack of a clear understanding for how such assays should be utilized and what the reported results ultimately indicate or, perhaps more importantly, what they do not indicate. Here, we provide a brief summary of the performance of a number of serologic assays reported in the literature, comment on what we do and do not know regarding our immune response to SARS-CoV-2, and provide a number of scenarios for which serologic testing will play a role during our global response to this pandemic.

## Abstract (precis)

The emergence of severe acute respiratory syndrome coronavirus 2 (SARS-CoV-2) brought with it rapid development of both molecular and serologic assays for identification of COVID-19 infections. While Food and Drug Administration (FDA) emergency use authorization (EUA) is required for clinical application of SARS-CoV-2 molecular tests, submission for EUA is currently a voluntary process for manufacturers of serologic assays. The absence of FDA oversight of serologic tests is concerning given that the commercially available serologic assays are highly variable, differing in their format, the antibody class detected, the targeted antigen, and the acceptable specimen types.

## TEXT

Shortly after its emergence in December 2019, the outbreak of severe acute respiratory syndrome coronavirus 2 (SARS-CoV-2) was declared a pandemic in March 2020 by the World Health Organization. A betacoronavirus, SARS-CoV-2 is the seventh member of the *Coronaviridae* family of viruses and is the causative agent of coronavirus disease 2019 (COVID-19) in humans (1). Given the acute and rapid onset of COVID-19, molecular testing of respiratory tract sample(s) to detect SARS-CoV-2 RNA remains the preferred diagnostic test for assessment of symptomatic patients who meet COVID-19 testing criteria as defined by the Centers for Disease Control and Prevention (CDC) and/or state and local health departments (2). In addition to molecular testing, there is increasing interest for use of serologic assays to detect antibodies against SARS-CoV-2. Unlike molecular testing, detection of an immune response to the virus is an indirect marker of infection. As such, development of robust serologic tests, alongside guidelines for appropriate utilization and interpretation relative to clinical and epidemiological needs, is essential to maintain safe patient care standards and support ongoing public health efforts.

Currently, over 91 manufacturers have notified the Food and Drug Administration (FDA) that they are offering internally validated serologic tests for commercial use, and at the time of this writing (17 April 2020), four products have received FDA emergency use authorization (EUA) (3, 4). Unlike prior public health emergencies, the FDA has indicated that EUA is not required for distribution or use of commercially available or laboratory-developed SARS-CoV-2 serologic tests. Rather, they require that laboratories validate the assays as they deem appropriate and notify the FDA of their use alongside inclusion of specific report comments outlining the limitations of these tests (3). The absence of FDA oversight of serologic tests is concerning given that the commercially available serologic assays are highly variable, differing in their format (e.g., lateral flow immunoassays [LFAs], enzyme-linked immunosorbent assays [ELISAs], and chemiluminescent immunoassays [CLIA]), the antibody class(es) detected (i.e., IgA, IgM, IgG, or IgM/IgG total), the SARS-CoV-2 antigen(s) used to design the assay (e.g., recombinant nucleocapsid protein [NP], subunit 1 of the spike glycoprotein [S1], the Spike glycoprotein receptor binding domain [RBD], etc.), and the acceptable specimen type (i.e., serum, plasma, whole blood, finger-stick whole blood). Given these differences in assay format and design, as well as a dearth of peer-reviewed data on performance characteristics, it is critical that laboratories considering serologic testing for SARS-CoV-2 perform a rigorous verification study to ensure the analytical performance and clinical accuracy of test results.

Such validations must include assessment of specificity using samples collected prior to or soon after the start of the outbreak from both healthy individuals and those with antibodies to other common infectious pathogens and from noninfectious disease etiologies. Most concerns regarding SARS-CoV-2 serologic assay specificity revolve around the potential for cross-reactivity with antibodies to the commonly circulating alpha- (NL63 and 229E) and beta- (OC43 and HKU1) coronaviruses (CoVs). Prior seroprevalence studies indicate that over 90% of adults age 50 and older have antibodies to all four common circulating CoVs; therefore, the potential for cross-reactivity in SARS-CoV-2 serologic assays is significant (5). Analysis of the amino acid sequence homology for both the NP and S1 proteins, common antibody targets in commercially available serologic tests, shows less than 30% similarity between the respective homologs found in SARS-CoV-2 and the commonly circulating CoVs (6, 7). Although this in no way rules out the potential for cross-reactivity, for comparison, SARS-CoV-2 and SARS share over 90% homology at the amino acid level. Interestingly, recent preliminary studies by multiple groups have shown limited to no cross-reactivity of antibodies to NL63, 229E, OC42, and HKU1 coronaviruses against recombinant forms of SARS-CoV-2 NP and RBD proteins by Western blotting or ELISA analysis (7, 8). However, due to the absence of thorough specificity data, the FDA currently requires inclusion of a comment indicating that false positive SARS-CoV-2 serologic test results may occur in patients with antibodies to non-SARS-CoV-2 coronaviruses (3). With respect to sensitivity studies, given our still emerging understanding of the kinetics of the immune response and antibody dynamics against SARS-CoV-2, serologic test kits would ideally be evaluated using serially collected serum samples from COVID-19 patients previously confirmed by a molecular assay or serum samples collected at a known time post-symptom onset (PSO). The resulting information would allow laboratorians to provide clinicians preliminary guidance with respect to timing of seroconversion relative to symptom onset, which due to the variety of serologic assays available, may be specific to the particular test used in the laboratory.

As laboratorians consider the need for SARS-CoV-2 serologic testing, among the first questions that likely arise are the following: “How well do serologic tests for SARS-CoV-2 antibodies actually work?” and “How will a SARS-CoV-2 serologic test result really be used in the clinical practice?” Unfortunately, the answers to some of these questions remain challenging to define, largely due to the limited peer-reviewed literature on serologic testing currently available. Generally, however, serologic assays are not relied upon for the diagnosis of acute viral respiratory tract infections—the rapid disease onset, often prior to the development of an immune response, and the availability of sensitive molecular diagnostics typically obviate reliance on antibody testing. Recent studies have evaluated the potential role of IgM antibodies against SARS-CoV-2 as a marker of recent infection. Among those, using an internally developed ELISA with recombinant SARS-CoV-2 NP antigen, Guo and colleagues recently showed that IgM antibodies were detectable in 85% of COVID-19-confirmed patients 1 to 7 days PSO (7). Importantly, however, they state that molecular testing remains preferred, with higher sensitivity during the first 5.5 days after illness onset, and conclude that IgM against SARS-CoV-2 may be useful in suspected COVID-19 patients negative by molecular methods after this time point. In stark contrast, albeit not yet peer-reviewed, another study evaluating a magnetic CLIA against the same NP antigen, showed 12% to 40% IgM seroconversion during the same time frame post onset (9). Using an ELISA designed to detect IgM antibodies against the RBD of the S1 subunit of the SARS-CoV-2 spike glycoprotein, data from Zhao et al. indicate that only approximately 28% of patients seroconvert to IgM positive by day 7 PSO, whereas 73% are positive by day 14 (10). In addition to IgM-based SARS-CoV-2 serologic assays, at least one immunologic assay to detect IgA class antibodies against SARS-CoV-2 is also commercially available. IgA antibodies are the most abundant immunoglobulins in mucosal surfaces, playing an essential role in protective immunity via toxin- and viral-neutralizing activities in the respiratory and gastrointestinal tracts (11, 12). Similar to IgM, recent studies show that IgA antibodies against SARS-CoV-2 are detectable as early as 1 day after symptom onset (7). The specificity of IgA-based assays has not yet been well vetted in the literature, however. To date, a preprint study concluded that despite higher sensitivity soon after infection, IgA specificity was lower compared to IgG-based tests, an observation that has been mirrored in unpublished studies by an author of this commentary (E. S. Theel, P. Slev, and S. Wheeler, unpublished data) (6). Finally, assessment of IgM and IgA antibody responses in patients infected with SARS virus showed that these two antibody classes did not provide earlier evidence of infection compared to IgG antibody testing (13). Collectively, the data presented in these initial studies and prior findings with SARS suggest that results from SARS-CoV-2 IgM and IgA serologic tests, if used, should be interpreted with significant caution until more robust performance characteristic and utilization studies are available.

In contrast to IgM and IgA class antibodies, detection of IgG antibodies against SARS-CoV-2 may have a larger role to play during this pandemic. Compared with other antibody classes, IgG is a longer lasting antibody and, similar to IgA, is associated with viral neutralizing activity, which is likely essential for recovery from COVID-19 (11, 14). Preliminary data suggest that IgG developed against different SARS-CoV-2 antigens becomes detectable in immunocompetent patients after at least 8 days PSO, with over 90% of individuals seropositive after day 14 of illness, although some individuals may take longer to seroconvert depending on their immune status or may never seroconvert if significantly immunosuppressed (9, 10). Although limited in breadth and not all yet peer-reviewed, initial studies suggest fairly high specificity (>95%) for IgG-based SARS-CoV-2 serologic assays against commonly circulating coronaviruses and other infectious pathogens (8, 9). Also, according to one reputable ELISA manufacturer, the false positivity rate observed with their SARS-CoV-2 S1-based IgG ELISA was 2.5% in serum samples positive for a diverse range of autoantibodies and 3.4% in serum samples from influenza vaccine recipients—such antibodies are not uncommon in the U.S. population. Importantly, true specificity studies require head-to-head comparison of commercially available serologic assays with neutralizing antibody tests, which are not widely accessible given the challenges of performing such assays. Currently, all available IgG serologic assays for SARS-CoV-2 are either qualitative or semiquantitative in design. For well-vetted assays, a negative result may indicate either no prior exposure or, for samples collected too soon after illness onset or from immunosuppressed patients, the absence of an as of yet detectable immune response. In contrast, a positive SARS-CoV-2 IgG result implies infection with the virus at some point in the recent or remote past. Importantly, however, the presence of SARS-CoV-2 IgG does not equate to protective immunity against reinfection nor does it indicate whether a patient has stopped shedding virus. In theory, seropositive individuals are expected to be at lower risk for reinfection than seronegative persons; however, neither the level nor the duration of protective immunity against COVID-19 is currently known. The potential for at least short-term immunity to COVID-19 is not unfounded, however. From prior immunity studies in recovered SARS patients, we know that neutralizing antibodies were detectable in 89% of patients up to 2 years after infection, with IgG antibodies becoming undetectable at 6 years (15, 16). Additionally, although not yet peer-reviewed, preliminary SARS-CoV-2 challenge studies in COVID-19 recovered adult rhesus macaques suggest that primary infection leads to protective immunity for at least 1 month post recovery (17). The true temporal duration of protective immunity to COVID-19, partial or otherwise, will take time to establish.

The reference standard method for detection of neutralizing antibodies, which may be used as a correlate of protective immunity, remains plaque reduction neutralization tests (PRNTs). These tests are not routinely performed in clinical laboratories, however, as they involve live viral culture, which for SARS-CoV-2 requires biosafety level 3 (BSL3) containment facilities, are laborious, dependent on a high level of expertise, and are not amenable to automation. Although alternative BSL2 protocols using pseudotyped vesicular stomatitis virus (VSV) expressing different SARS-CoV-2 surface antigens are being developed to obviate culture of live SARS-CoV-2, these methods remain in the research arena (18). Importantly, regardless of which neutralizing antibody test is being performed, it remains unclear what minimal neutralizing antibody titer correlates with protective immunity and whether results from the commercially available SARS-CoV-2 serologic assays can predict such immunity. Despite these significant unknowns, there remains interest and even demand to perform serologic tests at a national scale, with the potential to make consequential decisions based on the reported results.

The following are scenarios for which SARS-CoV-2 serologic testing, specifically IgG-based assays, may be useful given our current knowledge of the virus, our limited understanding of the immune response to it, and the urgent need for improved antiviral therapies and preventive measures.

## 

### Screening of recovered COVID-19 patients for convalescent plasma therapy.

Currently, among the most advocated patient-centered uses of SARS-CoV-2 serologic testing is for screening of COVID-19 recovered patients for the presence of anti-SARS-CoV-2 antibodies. If present, COVID-19 convalescent plasma (CCP) collected from these donors may be used to treat acutely ill patients with COVID-19 (19). Clinical trials are currently ongoing across the nation to evaluate the efficacy of convalescent plasma therapy in both sick patients and as potential postexposure prophylaxis of health care workers (HCWs) (www.ccpp19.org). Notably, the FDA investigational drug (IND) use requirements for these clinical trials, or for emergency IND use, indicate that donor convalescent plasma should have a neutralizing antibody titer of at least 1:160, although a titer of 1:80 is acceptable in the absence of other plasma (20). Unfortunately, neutralizing antibody tests are not widely available, and results from commercially available serologic assays are not known to correlate to neutralizing antibody titers. Given the urgent need of convalescent plasma as a potential bridging therapy until more targeted treatments or preventative measures are available, validated SARS-CoV-2 IgG serologic assays may be used to rapidly screen potential donors for the presence or absence of antibodies, with the goal of subsequently testing positive samples by neutralization assays. Studies are also ongoing to determine whether the semiquantitative results from a number of SARS-CoV-2 IgG ELISAs show any correlation to neutralizing antibody levels. Notably, recent studies on this topic have shown variable correlation between IgG detected by ELISA or immunofluorescence assays and PRNT. Overall, these data indicate that additional studies are needed to determine whether correlation between routinely available commercial assays and neutralizing antibody titers will be possible and whether it is assay dependent (6, 21, 30).

### SARS-CoV-2 seroprevalence studies.

Serologic testing to detect IgG class antibodies against SARS-CoV-2 will play an essential role in determining the true prevalence of this virus. This is particularly true if one considers the constant discussions around positive and negative predictive values of molecular tests for SARS-CoV-2. A prevalence of total disease in the community needs to be established in order to perform such calculations with any meaning. Given that the rate of asymptomatic infection with SARS-CoV-2 continues to be refined, with previously reported rates ranging from 4% to 80% across different populations and exposure scenarios, such seroprevalence studies will allow us to establish a more accurate regional or national denominator for the number of infected individuals, which will ultimately help to determine a true case fatality rate (22–25). Importantly, however, the serologic assay(s) utilized for such seroprevalence studies must exhibit exceptionally high specificity (≥97%) given that the prevalence of SARS-CoV-2 infection in the United States is likely still fairly low and the potential impact of cross-reactive antibodies to other circulating CoVs; a test with lower specificity could create significant bias and high rates of false positive results in large-scale serosurveys. Carefully designed serial seroprevalence studies, performed over time and including large cohorts, will also provide us with a better understanding of transmission patterns and may help determine when (or if) we reach a state of herd immunity. Herd (population) immunity occurs when a sufficient proportion of the population becomes immune to the infectious agent, thus limiting the chance for further infections to occur. The percentage of individuals that must be immune for this to occur depends on multiple factors, including the infectiousness or transmissibility of the infectious agent—the more transmissible the agent, the higher the percentage of the population that needs to be immune for herd immunity to be effective. The precise threshold for what percentage of the population would need to be immune to SARS-CoV-2 for this to occur is currently undefined; however, assuming that the SARS-CoV-2 basic reproductive number (*R*_0_) ranges from 2 to 3.5, this threshold may range from 40% to 75% (26). It is paramount to note, however, that given the early and intense social distancing measures instituted by federal and local governments, viral transmission has likely significantly decreased to the point that the actual herd immunity may not be achieved until such public health measures are lifted. Once available, a safe and efficacious vaccine should be able to induce widespread, population-level immunity.

### Monitoring immune responses to COVID-19 vaccine candidates.

The most recent reports indicate that there are over 100 SARS-CoV-2 vaccine candidates either in development, in initial preclinical stages, or which have entered human clinical trials (27). At least five of these are currently in phase 1 clinical trials and vary in their design, ranging from the use of lipid nanoparticles expressing the SARS-CoV-2 spike glycoprotein to modified dendritic cells expressing synthetic minigenes from selected viral proteins. Serologic testing for SARS-CoV-2 will play an important role for prescreening individuals prior to admission into vaccine clinical trials and to monitor the temporal immune responses in vaccine recipients and ultimately help to define vaccine efficacy. It is important to note that serological assays able to detect a neutralizing antibody response (i.e., PRNT) will be critical to provide the most accurate results for vaccine immunogenicity trials. Notably, whether such antibodies would potentially mediate antibody-dependent enhancement leading to adverse events is an important question that will be addressed through efficacy trials and postvaccine surveillance.

## SUMMARY

As a result of the novelty of SARS-CoV-2 and the limited data currently available regarding our immune response to it, well-vetted utilization strategies for SARS-CoV-2 serologic assays are lacking. Use of anti-SARS-CoV-2 antibody tests performed at a population-level to guide return-to-work decisions or to “restart the economy” is a topic of widespread discussion at the local, state, and national levels. Undeniably, this is an intriguing concept, with mass serologic screening potentially achievable at a national scale. However, we must remain cognizant of the current challenges and limitations of such an approach. First, there remains significant concern among laboratorians with respect to the over 91 serologic tests that are currently commercially available, for which the performance characteristics are not yet known. In fact, reports of poorly performing serologic tests are already emerging in the media (28). Should mass screening be recommended at the state or national level, it is imperative that data-based guidance regarding serologic test accuracy is available to laboratories considering such testing. Second, as outlined above, although a positive SARS-CoV-2 IgG result suggests prior infection with the virus, it does not independently imply protective immunity. Similarly, the duration of such immunity remains unknown. Finally, depending on the timing of SARS-CoV-2 infection and sampling for serologic testing, recently infected individuals may be IgG positive yet still be shedding virus as determined by molecular assays. Whether the detected viral RNA in these individuals equates to transmissible virus cannot be resolved without viral culture of the specimen at BSL3 containment—a method not available in clinical laboratories. Notably, a recent small study in hospitalized patients showed that infectious virus was not detectable in culture from seroconverted patients 8 days after symptom onset, whereas molecular testing of nasopharyngeal swab specimens remained positive beyond 14 days for most patients, suggesting that detected RNA by these assays represents residual RNA from noninfectious virus (21). This study, however, was conducted using mildly symptomatic individuals. Given that severely ill individuals remain SARS-CoV-2 RNA positive for several weeks despite the appearance of neutralizing antibodies, further studies using viral culture are necessary to better determine the period of transmissibility (29).

In conclusion, the availability of serologic assays to detect antibodies against SARS-CoV-2 presents us with additional tools to use from our SARS-CoV-2 pandemic response toolbox. As we learn more about our immune response to SARS-CoV-2, its level and duration of protective immunity, and as we gain a better understanding of the advantages and limitations of commercially available serologic assays, more defined, patient-centered utilization guidelines will likely emerge. These tests may be useful from a public health, risk management, and academic perspective, but additional data are required to fully drive this response.
